# Comparative and Experimental Studies on the Genes Altered by Chronic Hypoxia in Human Brain Microendothelial Cells

**DOI:** 10.3389/fphys.2017.00365

**Published:** 2017-05-31

**Authors:** Eugenia Mata-Greenwood, Dipali Goyal, Ravi Goyal

**Affiliations:** ^1^Center for Perinatal Biology, School of Medicine, Loma Linda UniversityLoma Linda, CA, United States; ^2^Epigenuity LLCLoma Linda, CA, United States

**Keywords:** epigenetic, hypoxic acclimatization, DNA methylation, CpG islands, HBMEC

## Abstract

**Background :** Hypoxia inducible factor 1 alpha (HIF1A) is a master regulator of acute hypoxia; however, with chronic hypoxia, HIF1A levels return to the normoxic levels. Importantly, the genes that are involved in the cell survival and viability under chronic hypoxia are not known. Therefore, we tested the hypothesis that chronic hypoxia leads to the upregulation of a core group of genes with associated changes in the promoter DNA methylation that mediates the cell survival under hypoxia.

**Results :** We examined the effect of chronic hypoxia (3 days; 0.5% oxygen) on human brain micro endothelial cells (HBMEC) viability and apoptosis. Hypoxia caused a significant reduction in cell viability and an increase in apoptosis. Next, we examined chronic hypoxia associated changes in transcriptome and genome-wide promoter methylation. The data obtained was compared with 16 other microarray studies on chronic hypoxia. Nine genes were altered in response to chronic hypoxia in all 17 studies. Interestingly, HIF1A was not altered with chronic hypoxia in any of the studies. Furthermore, we compared our data to three other studies that identified HIF-responsive genes by various approaches. Only two genes were found to be HIF dependent. We silenced each of these 9 genes using CRISPR/Cas9 system. Downregulation of EGLN3 significantly increased the cell death under chronic hypoxia, whereas downregulation of ERO1L, ENO2, adrenomedullin, and spag4 reduced the cell death under hypoxia.

**Conclusions :** We provide a core group of genes that regulates cellular acclimatization under chronic hypoxic stress, and most of them are HIF independent.

## Introduction

Hypoxia is one of the most common and severe stressor to an organism homeostasis. It also is an important factor associated with the multitude of physiological (high altitude residence) and pathological conditions (congestive cardiac failure, pulmonary fibrosis, chronic anemia, cancer). Oxygen sensing is a property of essentially all cell types, and the response to hypoxia is multidimensional, involving complicated intracellular signal transduction networks (Semenza, 2001; Cummins and Taylor, 2005; Kaelin and Ratcliffe, 2008). Under limiting oxygen concentration, at the organismal level, others and we have observed fetal growth restriction (Goyal et al., 2011), and at the cellular level reduced viability (Su et al., 2015). Under hypoxic condition, cells undergo several changes to survive, and dysregulation of these changes makes them vulnerable to death (Zou et al., 2014). The mechanisms are not entirely known, however.

Studies have demonstrated that under normoxic conditions, prolyl hydroxylase dioxygenases (PHD) hydroxylate hypoxia inducible factor 1 alpha (HIF1A) and marks it for degradation (Ratcliffe et al., 1999). Under hypoxic conditions, PHD becomes deactivated and HIF1A accumulates (Semenza, 2001). HIF1A is a transcriptional regulator and its accumulation increases the expression of several genes involved in anaerobic glycolysis and those involved in reducing cellular oxygen consumption. As a result, there is a relative increase in cellular oxygen, which leads to reactivation of PHD and despite continued hypoxia, HIF1A returns to the basal (normoxic) levels (Ginouvous et al., 2008; Watson et al., 2009; Goyal and Longo, 2014). However, following the return of HIF1A to the normoxic levels in chronic hypoxia exposure, the cell is able to survive (Goyal and Longo, 2014). Again, the mechanisms are not known.

In the present study, we examined the effect of chronic hypoxia on cell viability and apoptosis of human brain micro endothelial cells (HBMEC). Also, we conducted a whole transcriptomic microarray to discover the changes with hypoxia in HBMEC. We further examined the genome-wide methylation changes in the same cells to examine associated methylation changes. We conducted a signal transduction pathway/networks analysis to identify pathways altered in response to chronic hypoxia. We also compared our data to more than 16 other microarray datasets available through GEO Database to identify a set of core genes, which are altered in response to hypoxia in all these studies. Furthermore, we mutated these genes using CRISPR/Cas9 to identify the crucial genes and their effect on cell viability and survival under hypoxic conditions. Overall, we attempted to identify mechanisms, which were responsible for cell acclimatization and survival under chronic hypoxic conditions.

## Materials and methods

Experiments were conducted on primary HBMECs obtained from the Applied Cell Biology Research Institute Cell Systems Repository (Kirkland, WA). All experiments were conducted on cells at passage 8. Cells were exposed to 0.5% oxygen for days 1 to 3. Hypoxia was administered in a hypoxic chamber using a ProOxC21 regulator (BioSpherix Inc. Lacona, NY).

### Cell validation and characterization

Beside observing cell morphology of endothelial cells (cobblestone appearance), immunohistochemistry (IHC) experiments were conducted to detect CD31 (Tachezy et al., 2010) and FITC conjugated Ulex europaeus I lectin antigen (Holthöfer et al., 1982), which are specific for endothelial cells. IHC experiments were conducted using CD31 antibodies obtained from Abcam Inc. (Cambridge, MA) and ulex antigen obtained from Sigma-Aldrich Co. LLC (St. Louis, MO). Additionally, we conducted *in-vitro* angiogenesis assay to validate functional endothelial cells by observing tube formation using matrigel (Corning Inc., Corning, NY).

### Viability and proliferation assay

For examining cell survival under hypoxic conditions, we used the Real-Time-Glo™ MT Cell Viability Assay using manufacturer's protocol (Promega Corporation, Madison, WI). Cells were cultured in several 96-well plates, and each experiment was repeated 4 times to examine repeatability and reproducibility.

### Cell apoptosis assay

For examining apoptosis, we used the Caspase-Glo 3/7 assay following manufacturer's instruction (Cat # G8090, Promega Corp.). This assay is a luminescent assay that measures caspase−3 and −7 activities. The assay provides a substrate, which glows following its cleavage by active caspases 3 or 7 (if present in the cells). Importantly, luminescence is proportional to the amount of caspase activity present. This apoptosis assay was conducted on cells exposed to 0.5% hypoxia for 72 h in biological replicates of 6.

### Microarray experiment

Microarrays were conducted on the biological replicates of 6 for each group at day 3 of 0.5% and 21% oxygen exposure to HBMEC cells. We have described these methods in our previous publications (Goyal et al., 2010, 2013; Goyal and Longo, 2012, 2014). Briefly, Agilent Human GE 8X60K V2 arrays (Design ID 039494) were obtained (Agilent Technologies, Santa Clara, CA) and analysis was conducted by utilizing commercial services (GenUs Biosystems, Northbrook, IL). RNA was isolated by the standard Trizol method and quantitated by UV spectrophotometry (OD260/280). RNAs with a value of OD260/280 between 1.8 and 2.0 were included in the study. The quality of the RNA was further assessed using an Agilent Bioanalyzer (Supplementary Figure 1). Labeled cRNAs were prepared by linear amplification of the Poly(A)+ RNA population within the total RNA sample. Briefly, 1 μg of total RNA was primed with the T7 RNA polymerase promoter with a d(T)24 sequence containing DNA oligonucleotides and then reverse transcribed. After second-strand cDNA synthesis and purification of double-stranded cDNA, we conducted *in vitro* transcription using T7 RNA polymerase. The labeled cRNA quantity and quality were examined by spectrophotometry and the Agilent Bioanalyzer (Supplementary Figure 2). We then fragmented 1 μg of purified cRNA to uniform size and applied to the microarray chips in hybridization buffer. Processed arrays were hybridized at 65°C for 17 h in a shaking incubator and washed at 37°C for 1 min. The processed arrays were then scanned at a 5 μm resolution with an Agilent G2565 Microarray Scanner (Agilent Technologies). We used Agilent Feature Extraction software to process the scanned images from arrays for gridding and feature intensity extraction, and GeneSpring GX v7.3.1 software (Agilent Technologies) was used to analyze the data. Supplementary Figure 3 demonstrates the probes with intensity above background in at least 4 replicates following hypoxia treatment (21,221–21,938 probes). To further demonstrate the successful clustering of samples (control vs. hypoxia) differentially expressed genes (>2-folds, *p*-value < 0.05) were normalized to the median expression across the 12 samples and plotted on cluster tree analysis (Supplementary Figure S4).

The annotated genes were analyzed using the Ingenuity Pathway Analysis Program (IPA; Ingenuity Systems, Redwood City, CA). We conducted the IPA downstream effects analysis to predict an activation (increase) or an inactivation (decrease) in downstream molecular activities occurring in the tissues being studied. Based on the published studies (indexed in Pubmed), the expected effects of the gene (upregulation or downregulation) on a particular biological process was compared for the observed changes in the mRNA expression in the present study. The analysis determines how many known targets of each transcription regulator are present in the user's dataset, and compares their direction of change (upregulated or downregulated) to what is expected from the literature. If the observed direction of change in the gene expression is mostly consistent with the literature, then a prediction is made about that activation state (“activated” or “inhibited”). For each potential transcriptional regulator (“TR”) we computed two statistical measures, an overlap *p*-value and an activation z-score (Krämer et al., 2014). The purpose of the overlap *p*-value was to identify transcriptional regulators that are able to explain observed gene expression changes. It was calculated using Fisher's Exact Test, and significance is attributed to *p*-values < 0.01. The primary purpose of the activation z-score was to infer the activation states of predicted transcriptional regulators. However, genes can be regulated by a number of upstream regulators with differing effects on the activation state, and it is not known which will dominate in a particular system. Therefore, we took a statistical approach and defined a quantity (z-score) that determines whether an upstream transcription regulator has significantly more “activated” predictions (*z* > 0) or more “inhibited” predictions (*z* < 0). Significant z-score means that we reject the hypothesis that predictions are random with equal probability. Additionally, the ratio of the total genes altered in the present system to the total genes known to be the part of a particular pathway was determined.

### Genome-wide methylation analysis

Methylation analysis was conducted at the University of Southern California Epigenomic Core. The DNA was extracted using a chloroform based extraction method and was bisulphite modified using the EZ DNA Methylation Kit D5008 (Zymo Research, Orange, CA) according to the manufacturer's instructions. Average DNA concentration was ~62 ng/μL. For methylation analysis, we used Illumina Infinium Human Methylation 450K BeadChip Array. Bisulphite converted DNA was amplified, fragmented, and hybridized to the array chips (each chip accommodates 12 samples). We then performed a single base extension using labeled DNP- and biotin-labeled dNTPs. The arrays were imaged using a Bead Array Reader and processed for intensity data extraction according to the manufacturer's instructions. On the chip, each CpG locus was represented by two specific oligomers, one representing methylated DNA (*M*) and the other for unmethylated DNA (*U*). The methylation status of a specific CpG site is then calculated from the intensity values of the *M* and *U* alleles, as the fraction of fluorescent signals ß = [*M/(M*+*U*+*100)*]. ß-values were continuous variables between 0 (absent methylation) and 1 (completely methylated) representing the fraction of combined locus intensity.

### Clustered regularly interspaced short palindromic repeats type II system (CRISPR/Cas9)-mediated gene editing

Gene silencing experiments were conducted using CRISPR/Cas9 technology. CRISPR/Cas9 consists of two components: a “guide” RNA (gRNA) and a non-specific CRISPR-associated endonuclease (Cas9). The gRNA is a short synthetic RNA composed of a “scaffold” sequence necessary for Cas9 (endonuclease)-binding gene editing. The sequences of gRNA used to target the 9 genes which produced more than 2-fold knockdown are provided in Supplementary Table 1. We generated lentivirus using third generation packaging plasmids, and to confirm the gene knockdown, we conducted a High-Resolution Melting (HRM) Curve analysis to detect wild type vs. mutated transcripts. A representative HRM curve of EGLN3 mutation following CRIPR/Cas9 experiment is provided in Supplementary Figure 5. Following mutation, the knockdown of mRNA levels was validated by real-time PCR; cells with knockdown of more than 2-fold were included in the downstream experiments and analysis.

### Real-time PCR validation

To validate the results of the microarray analysis, we chose top-five upregulated and downregulated genes. We designed primers with the use of Primer 3 web-based software (http://frodo.wi.mit.edu/primer3/) using the same probe sequences as those on the microarray chip. The primers (Supplementary Table 2) were synthesized by Integrated DNA technologies (Coralville, CA). We reverse transcribed total RNA (1 ug per reaction) using Quantitect reverse transcriptase kit (Qiagen, Valencia, CA). Relative expression was normalized to 18S RNA and fold-changes were calculated using the ΔΔCt method with normalization of individual PCR efficiencies (Ramakers et al., 2003). Samples were analyzed on the Rotor-Gene Q (Qiagen Inc.).

### Statistics

For all the experiments “*n*” was 6 in each group. For cell viability and apoptosis assay, we used one-way ANOVA with *post-hoc* Bonferroni's test to determine significant changes in the mean value. For microarray analysis, we normalized the raw intensity data from each gene to the 75th percentile intensity of each array to compare individual gene expression values across arrays. The genes with values higher than background intensity for all samples within each group were considered for downstream analysis. Differentially expressed genes were identified by 2-fold change and Welch *T*-test *p*-values < 0.05 between each treatment group and its normoxic control. Statistical significance in the real-time PCR data was determined by Student's *T*-Test.

## Results

### Characterization of endothelial cells

We characterized the cells to determine if the cells are endothelial in origin (Supplementary Figure 6). Supplementary Figure 6A demonstrates control endothelial cells morphology. Supplementary Figure 6B demonstrates capillary tube formation on plating the endothelial cells on Matrigel. Supplementary Figure 6C demonstrates the green staining of HBMEC to FITC conjugated Ulex Lectin antigen, and Supplementary Figure 6D shows red staining to CD31 antigen and blue staining nuclei. These experiments conclusively prove that these were endothelial cell type.

### Chronic hypoxia reduced HBMEC viability

**Figure 1:**
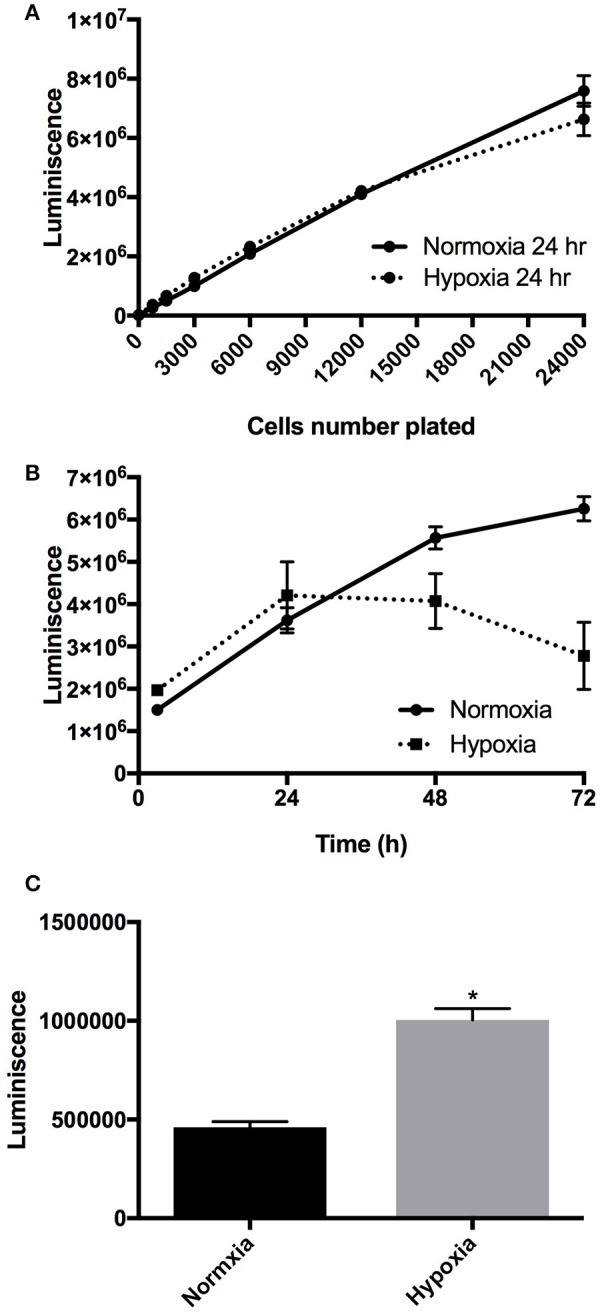
**(A)** Demonstrates the luminescence detected by real-time cell viability assay with a different number of cells/well with 24 h of normoxia and hypoxia exposure. **(B)** Demonstrates the changes in luminescence during 1 to 3 days of hypoxia exposure indicating the changes in cell viability and proliferation. *N* = 6 in each group. Data is presented as mean and standard error of mean. **(C)** Demonstrates a significant increase (*P* < 0.05; *n* = 6) in Caspase 3/7 activity in hypoxic cells as compared to that from normoxic cell. *N* = 6 in each group; ^*^Denotes *P* < 0.05. Data is presented as mean and standard error of mean.

We conducted cell viability assay using RealTime-Glo™ MT Cell Viability Assay using manufacturer's protocol (Promega Corporation, Madison, WI) for examining cell survival under hypoxic conditions (0.5% oxygen for 72 h). We plated the cells at increasing density (750, 1,500, 3,000, 6,000, 12,000, and 24,000 cells per well) in a 96-wells plate. Each assay was conducted in 8 replicates and three different plates at three different times. Figure 1A demonstrates the luminescence detected with a different number of cells. As shown in the figure, the real-time cell viability assay was able to detect linearly up to 24,000 cells per well. Next, we measured the effect of hypoxia (1 to 3 days) on cell viability and proliferation. We observed that exposure of HBMEC cells to 0.5% oxygen significantly increased cell death, as compared to control (Figure 1B). To confirm the effect of hypoxia on cell survival and viability, we examined the effect of 0.5% oxygen for 72 h on cellular apoptosis. Figure 1C demonstrates a significant increase (*P* < 0.05; *n* = 6) in caspase 3/7 activity in hypoxic cells as compared to that of the normoxic cell. To examine the mechanisms of hypoxia-induced apoptosis, we conducted oligonucleotide microarray experiment.

### Chronic hypoxia and gene expression

**Figure 2:**
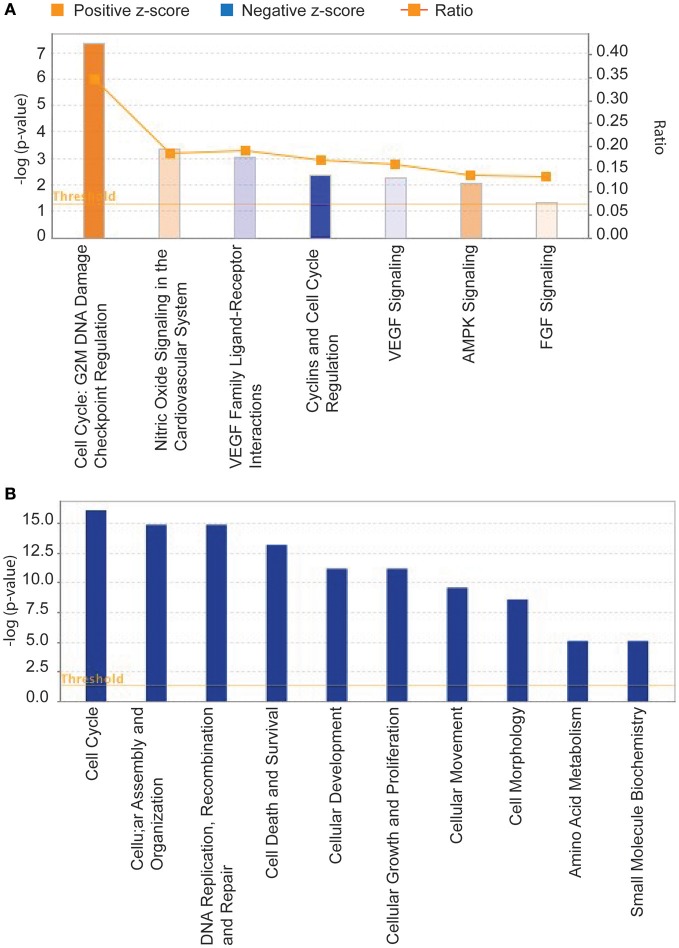
Demonstrates the chief canonical pathways **(A)** and the chief functional pathways **(B)** identified by Ingenuity Pathway Analysis on genes altered by exposure to 0.5% oxygen (hypoxic) and 21% oxygen (normoxic) for 3 days. *N* = 6 in each group.

In response to chronic hypoxia, 1,040 and 713 genes were 2-fold (*P* < 0.05) upregulated or downregulated, respectively. On analysis with Ingenuity Pathway Analysis, we observed the top canonical pathways altered included cell cycle checkpoint regulation, nitric oxide signaling and VEGF signaling (Figure 2A) and the chief cellular function affected were cell cycle, cellular assembly and organization, cell death and survival (Figure 2B). The molecules most affected included PTGIS, IGFBP3, adrenomedullin and other. The top downregulated molecules included, PRSS35, FABP4, etc. A complete list of upregulated genes is provided in the Supplementary Table 3, and downregulated genes are provided in the Supplementary Table 4. We then compared the genes altered in the present study with 16 other microarray studies on different cells. We identified 9 genes common to the 17-microarray studies including the present study (Table 1).

**Table 1:** List of core genes altered in response to hypoxia in different microarray analysis.

**Accession no**.		**GDS3483**	**GSE3045**	**GDS3483**	**GDS3524**	**GSE4725**	**GDS2902**	**GSE35932**	**GDS2951**	**GDS2760**	**GSE4725**	**GSE35932**	**GSE35932**	**GDS1648**	**GSE35932**	**GSE35932**	**GSE35932**
Days of hypoxia	5 Days	2 Days	2 Days	2 Days	2 Days	2 Days	1 Day	1 Day	20 h	16 h	16 h	12 h	8 h	8 h	4 h	2 h	1 h
Cell type	HBMEC	CM	HUVEC	PVMEC	REC	ASMC	LEC	HUVEC	PBL	MCF4	ASMC	HUVEC	HUVEC	HC	HUVEC	HUVEC	HUVEC
**GENE SYMBOL**																	
ADM	4.39	1.67	4.72	2.92	4.92	5.99	3.53	13.42	1.88	60.26	1.77	10.82	9.75	24.44	5.93	3.46	1.5
EGLN3	10.31	1.87	96.57	2.96	1.59	0.45	33.86	13.01	1.19	15.59	2.22	9.75	6.84	NA	3.95	1.46	0.98
ENO2	2.74	1.95	4.72	2.68	3.62	1.85	5.91	17.64	1.23	22.20	2.91	8.45	13.37	1.08	3.31	0.8	0.94
ERO1L	2.83	2.22	2.24	1.64	2.41	4.31	2.13	2.63	1.78	6.62	7.85	1.72	1.34	2.47	1.54	0.98	0.73
LOX	5.72	16.96	2.46	1.64	3.50	1.72	3.89	12.31	0.90	19.06	1.36	5.75	2.96	1.13	1.84	1.06	0.94
P4HA1	4.14	1.75	3.43	1.67	3.42	1.85	2.66	8.90	3.70	5.30	1.80	4.95	4.13	1.13	2.04	1.14	1.05
PLOD2	2.67	2.43	1.83	1.94	2.10	3.46	2.51	3.65	0.91	2.62	2.97	2.56	2.72	NA	1.45	1.09	1.03
SLC2A3	2.75	1.66	3.51	2.68	4.49	3.01	4.05	6.53	2	1.80	1.95	6.29	4.67	1.46	2.56	1.2	0.78
SPAG4	2.66	1.72	4.80	1.51	2.99	2.41	1.65	23.63	0.87	22.99	1.51	12.50	7.75	NA	2.43	0.84	1.01

*HMBEC, human brain microendothelial cells; CM, cardiomyocytes; HUVEC, human umbilical vein cells; PVMEC, pulmonary vein microendothelial cells; REC, renal endothelial cells; ASMC, aortic smooth muscle cells; LEC, lymphatic endothelial cells; PBL, peripheral blood lymphocytes; MCF4, breast carcinoma cell line; HC, primary hepatocytes; NR, Data not reported*.

### Chronic hypoxia and HIF1A mRNA expression

Previously, others and we have demonstrated that with chronic hypoxia (≥ 2 days) HIF1A levels were equivalent to those of normoxic (Ginouvous et al., 2008; Goyal and Longo, 2014). However, studies have demonstrated that HIF1A upregulation is critical for cell survival in during first 24 h (Tanaka et al., 2011). To identify the known HIF-responsive genes, we analyzed data from three different studies, which conducted an integrative genome-wide expression and chromatin immunoprecipitation using HIF antibodies, and/or computational strategies with experimental validation (Benita et al., 2009; Mole et al., 2009; Ortiz-Barahona et al., 2010). In the first study, the investigators examined 19 microarray datasets (obtained from GEO and ArrayExpress repositories) and ranked the genes based on their response to hypoxia and HIF-binding sites (Benita et al., 2009). This study identified 500 genes, which may be the target of HIF. In the second study, the investigators conducted genome-wide chromatin immunoprecipitation using antibodies to two major HIFA subunits (HIF1A and HIF2A), and correlated the results with genome-wide transcript profiling (Mole et al., 2009). In the third study, the investigators conducted phylogenetic foot printing and transcription profiling meta-analysis to identify HIF-target genes (Ortiz-Barahona et al., 2010). This study identified 216 genes, which may be the targets of HIF. On comparison of these three studies, we observed only 19 genes common to all the three studies (Supplementary Table 5). Interestingly, only two of the 19 genes were also present among the 9 genes common to the 17-microarray studies including the present study (Table 2). These were endoplasmic reticulum oxidoreductin like protien 1 (ERO1L) and procollagen-lysine 2-oxoglutarate 5-dioxygenase 2 (PLOD2). The other seven genes that were common to all chronic hypoxia studies are not HIF1A dependent.

**Table 2:** Enlist the presence (P) or absence (Ab) of the core hypoxic genes in the three studies on HIF1A target identification.

	**Benita et al**	**Mole et al**	**Ortiz-Barahona et al**
ADM	Ab	Ab	P
EGLN3	P	Ab	P
ENO2	P	P	Ab
ERO1L	P	P	P
LOX	P	Ab	P
P4HA1	P	Ab	P
PLOD2	P	P	P
SLC2A3	Ab	Ab	Ab
SPAG4	P	Ab	Ab

### Hypoxia and DNA methylation

Because with prolonged hypoxia, HIF levels return to normal, we hypothesized that HIFA leads to changes in DNA methylation, which leads to changes in chronic hypoxia associated transcriptome. To test this hypothesis, we conducted a genome-wide methylation analysis using the Illumina Infinium HumanMethylation450 (450K) BeadChip. We calculated the Beta value (Intensity of Methylated CpG/(Intensity of Methylated CpG+Intensity of Unmethylated CpG+100) for each probe in 8 control and 8 hypoxic samples. In response to chronic hypoxia 356 and 16 genes were >1.5-fold (*P* < 0.05) hypermethylated (Supplementary Table 6) or hypomethylated (Supplementary Table 7). On comparing the genes with altered methylation and mRNA, we observed that 17 downregulated genes (Table 3) were associated with significant hypermethylation in the promoter regions. Notably, none of the upregulated genes were associated with significant hypomethylation. Apparently, chronic hypoxia-mediated gene upregulation is not dependent on DNA methylation.

**Table 3:** Shows the genes with correlative changes in mRNA levels and DNA methylation in response to hypoxia.

**Gene symbol**	**mRNA ratio hypoxia/control**	**Methylation ratio hypoxia/control**
MYBL2	0.18	1.52
MFSD2A	0.23	1.57
NCAPG	0.24	1.52
C17orf53	0.25	1.58
HELLS	0.25	1.52
MAD2L1	0.25	2.45
KIFC1	0.25	1.58
NOS3	0.26	1.58
FAM111B	0.32	1.73
CIT	0.33	1.55
SFPQ	0.37	1.51
TXNDC11	0.37	1.51
FSTL5	0.44	1.63
ENSA	0.48	1.58
ZNF207	0.48	1.57
PGBD4	0.48	1.51
KPNA2	0.49	1.84

### Comparison of the present data with other studies

To identify the core genes that are altered in response to hypoxia, irrespective of the cell type, we compared the dataset of the present study with 16 other microarray studies examining cellular hypoxia. These studies were selected from the GEO Omnibus Repository. The studies included were restricted to human cells with oxygen levels between 0.5 and 1%. Studies, where CoCl2 or other chemicals were used to mimic hypoxia were excluded. We compared our data with human studies, which administered 0.5 to 1% hypoxia for different time durations starting from 1 to 48 h. Other than the present study there were three other studies with hypoxia of 48 h. We have identified 9 molecules which upregulated in all the studies (Table 1). Of note, none of the study (including the present study) with hypoxia more than 48 h demonstrated upregulation of HIF1A or HIF2A at the mRNA level. Also, none of the downregulated genes were common in all the studies examined.

### CRISPR/Cas9 mediated knockdown of the 9 common genes identified in comparative studies

**Figure 3:**
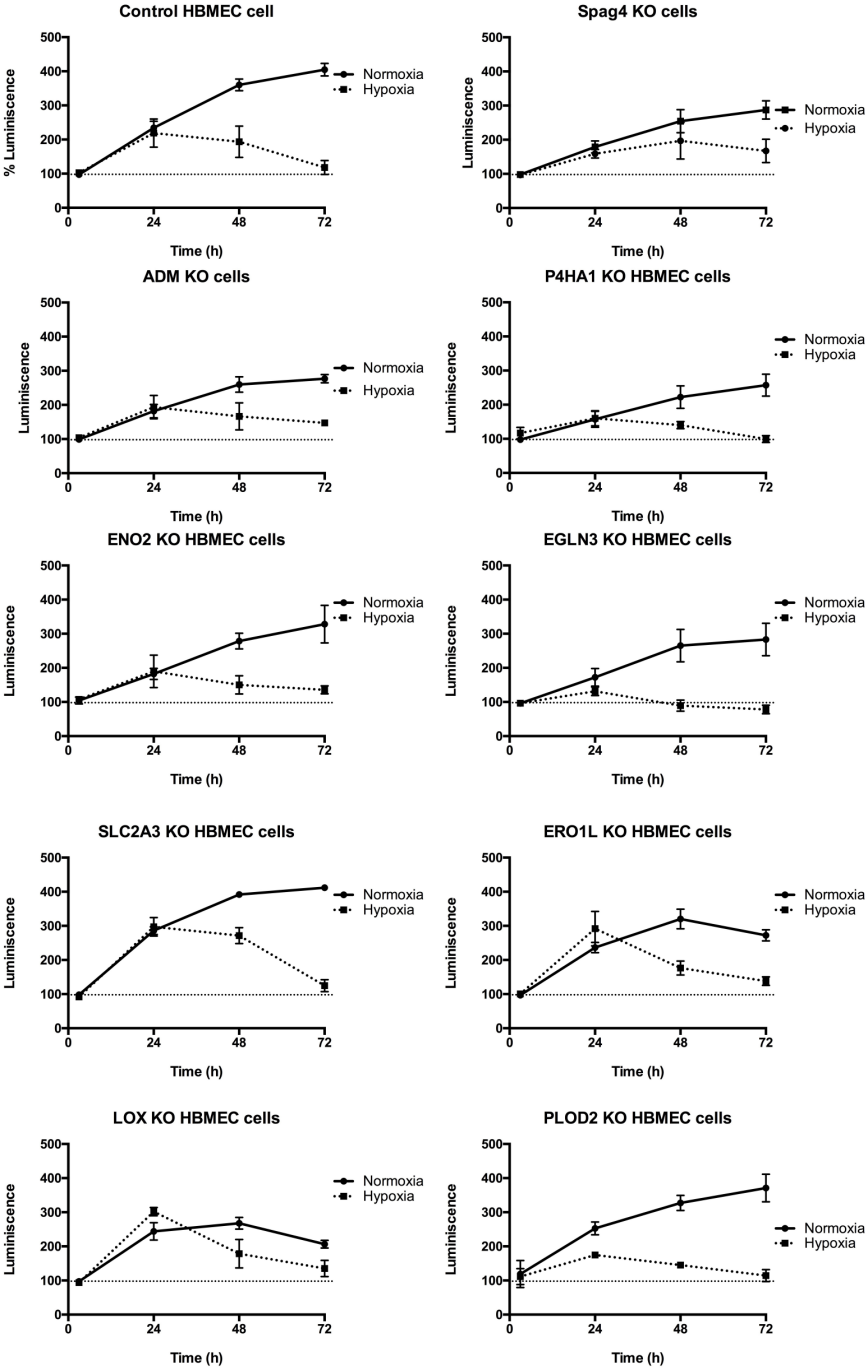
Demonstrates the effect of 0.5% oxygen (hypoxic) and 21% oxygen (normoxic) for 3 days on normal cells and the cells with mutated genes by Crispr/Cas9. *N* = 6 in each group.

To establish the functional significance of the 9 genes identified in the above-mentioned comparative studies, we conducted knockdown experiments using CRISPR/Cas9 technique. First, we validated significant knockdown by conducting real-time PCR. The guideRNA showing more than 2-fold knockdown were included in the further studies (Supplementary Table 1). As shown in Figure 3, we observed a significant cell death during 48 h under hypoxic conditions in EGLN3 and PLOD2 knockout cells (as compared to control cells). Importantly, at 72 h in adrenomedullin, ENO2, ERO1L and SPAG4 knockout cells significant increase in the cell viability as compared to the control cells (Table 4).

**Table 4:** Showing % Viability in control vs. knockout cells following 0.5% hypoxia exposure.

	**48 H**			**72 H**		
	**Mean viability (%)**	**SEM**	*P*-Value^*^	**Mean viability (%)**	**SEM**	*P*-Value^*^
Control (WT)	93.72	23.07		10.11	7.56	
ADM Knockout	66.49	19.8	0.40	47.27	5.22	0.01
EGLN3 Knockout	0	0	0	0	0	0
ENO2 Knockout	50.1	13.49	0.15	35.6	6.71	0.04
ERO1L Knockout	82.72	13.65	0.70	36.92	6.34	0.03
LOX Knockout	67.75	13.42	0.37	18.9	2.92	0.39
P4HA1 Knockout	40.26	6.06	0.11	0.50	5.15	0.29
PLOD2 Knockout	40.49	4.29	0.01	3.50	3.90	0.69
SLC2A3 Knockout	171.51	11.79	0.02	22.04	11.62	0.41
SPAG4 Knockout	96.95	26.68	0.93	67.29	17.16	0.02

* *P-value, compared to control hypoxic cells at the same time point*.

## Discussion

The key findings of the present study were: (1) The viability of endothelial cells reduced and apoptosis increased under hypoxic stress. (2) Microarray examination supports that hypoxic stress is associated with changes in gene expression responsible for cell cycle arrest and cell death. (3) Hypoxia-induced changes in gene upregulation do not correlate necessarily with changes in DNA methylation. (4) In response to chronic hypoxia, there was a core group of 9 genes, which were upregulated in several human studies. (5) Of these 9 genes, only two genes ERO1L and PLOD2 were HIF1A dependent, whereas, the other seven genes were not. (6) EGLN3 is crucial for cell survival under hypoxic conditions. (6) Knockdown of ERO1L and LOX may prevent hypoxia-induced cell death.

Hypoxia is a major stressor in an organisms' life and probably the most physiologically and clinically relevant stress. Of note, human development starts in a hypoxic environment which has been referred as “Mount Everest in Utero” (Eastman, 1954; Longo, 1987). Importantly, the effects of hypoxia are dependent on the severity of the hypoxic stress. Mild to moderate hypoxia may be beneficial in improving human health and may even prevent obesity and lower the mortality from coronary heart disease and stroke (Heinonen et al., 2016). However, at present, the definitions of mild, moderate, and severe hypoxia are not well defined. Evidence shows that the O_2_ levels in different human tissues are between 0.5 and 7% (Table 5). In the brain, under normal physiological conditions, depending on the depth of measurement the O_2_ levels were recorded between 0.55 and 8% (Table 4). Nonetheless, clinical conditions such as stroke, traumatic brain injury, skin wounds, and myocardial infarction result in severe hypoxic stress for the cell associated with the injury site and in the surrounding regions. Evidence suggests that in the brain regions associated with TBI the partial pressure of O_2_ can fall as low as 4 to 10 mm Hg (Dings et al., 1998; Hlatky et al., 2004) which corresponds to 0.5 to 1.5% O_2_ levels. For adequate healing of the injured tissue, it is critical for the endothelial cells to survive and create new blood vessels and provide oxygen. In the present study, we attempted to identify the genes which may prolong the survival of the brain microendothelial cells under severe hypoxic conditions and may provide better healing response following stroke or traumatic brain injury.

**Table 5:** Oxygen concentration in various body tissues.

	**Oxygen %**	**Oxygen partial pressure (mm Hg)**	**References**
Air	21.0	160	
Alveolus	14.4	110	
Arterial blood	13.1	100	
Venous blood	5.3	40	
Superficial skin	1.1	8	Wang et al., 2003
Dermal papillae	3.2	24	Wang et al., 2003
Sub-papillary plexus	4.6	35	Wang et al., 2003
Brain regions	0.55 to 8	4.1 to 60	Panchision, 2009
Brain – Duramater	6.3	48	Meixensberger et al., 1993
7–12 mm below duramater	4.3	33	Dings et al., 1998
17–22 mm below duramater	3.4	26	Dings et al., 1998
22–27 mm below duramater	3.2	24	Dings et al., 1998
Kidney – Cortex	6.6	50	Brezis and Rosen, 1995
Kidney medullary region	1.3 to 2.6	10 to 20	Brezis and Rosen, 1995
Medial head of gastrocnemius	3.8	28.9	Bylund-Fellenius et al., 1981
Vastas lateralis	4.2	32	Beerthuizen et al., 1989
Biceps	3.3	25	Boekstegers et al., 1990
Deltoid	4.5	34	Ikossi et al., 2006
Tibialis anterior	2.8	21	Kiaer and Kristensen, 1988
Bone marrow	6.7	51	Ishikawa and Ito, 1988
Bone marrow	7.2	55	Harrison et al., 2002
Human renal carcinoma	1.3	9.6	Lawrentschuk et al., 2005
Human liver tumor	0.8	6	Vaupel et al., 2007
Human primary brain tumors	1.7	13	Vaupel et al., 2007

It is well established that the transcription factors HIF1A and HIF2A are the key regulators responsible for the induction of genes that facilitate adaptation and survival of cells/organism from normoxia (21% O_2_) to hypoxia (1% O_2_; Wang and Semenza, 1993). Studies have established that under hypoxic conditions HIF1A and 2A bind to the hypoxic response element (HRE; 5′-A/GCGTG-3′) as a heterodimeric complex consisting of a subunit HIF-1α and HIF-1β (Wang et al., 1995). HIF-1β is also known as the aryl hydrocarbon nuclear translocator (ARNT), which was originally identified as a binding partner of the aryl hydrocarbon receptor (Hoffman et al., 1991). These proteins belong to the basic helix-loop-helix–Per-ARNT-Sim (bHLH–PAS) protein family (Wang et al., 1995). The bHLH and PAS motifs are required for heterodimer formation between the HIF-1α and HIF-1β subunits and binding to HRE. Two transactivation (stimulation of transcription) domains, N-terminal (N-TAD) and C-terminal (C-TAD) have been identified in the C-terminal of HIF1α (Ruas et al., 2002). Importantly, HIF-1α also contains an oxygen-dependent degradation domain (ODDD) that mediates oxygen-regulated stability (Pugh et al., 1997). HIF exists in three other isoforms HIF2α, HIF3α, and inhibitory PAS (IPAS). However, HIF1α is the major isoform expressed in most of the tissues.

Under normoxic conditions, O_2_ binds to EGLN proteins (containing a PHD) and activates them. Activated EGLN hydroxylates two proline residues (P402 and P564) of HIF1α, and following its hydroxylation HIF1α is recognized and degraded by Von Hippel-Lindau protein (pVHL) E3-ligase complex (Ratcliffe et al., 1999). However, in hypoxic conditions, EGLN dissociates from O_2_ and becomes deactivated and is unable to hydroxylate HIF1α. In the absence of hydroxylation, HIF1α is not recognized by degradation complex pVHL E3 ligase and accumulates. HIF1α then dimerizes with HIF1β and binds to the hypoxia response element (HRE; 5″A/GCGTG3″) sequence triggering transcription of a number of genes required for cell survival (Semenza, 2001).

In the present study, we observed that with chronic hypoxia HIF1A was significantly downregulated. Moreover, we observed that EGLN1 and EGLN3, which are downregulated in acute hypoxia (first 24 h), were upregulated with continued hypoxic exposure (for 72 h). Furthermore, HIF3A, which is a negative regulator of HIF1A and HIF2A (Heikkila et al., 2011), was also upregulated in the present study. Thus, acute hypoxia activates HIF pathway; however, the present study indicates that with chronic hypoxia HIF pathway is downregulated despite continued low oxygen levels. Similar findings have been demonstrated by others (Ginouvous et al., 2008). However, the mechanisms are not completely known. Furthermore, on analyzing different studies predicting HIFA targeting genes, there appears to be little consensus. On comparing three such studies that claimed to identify HIF1A targeting genes (Benita et al., 2009; Mole et al., 2009; Ortiz-Barahona et al., 2010), we found the overlap of only 19 genes among the three studies. Of these 19 genes, only 6 genes were present in our dataset of chronic hypoxia altered genes. This observation may indicate that chronic hypoxia induces different pathways than acute hypoxia in which HIFA plays a crucial role. At present, we do not know the molecules or pathways, which play a critical role in maintaining cell survival under chronic hypoxia. However, the present study identified nine key molecules associated with chronic hypoxia, which may be the successor of HIF pathway with continued hypoxia.

In the present study, we knockdown the 9 common genes (Spag4, adrenomedullin, P4HA1, ENO2, EGLN3, SLC2A3, ERO1L, LOX, PLOD2) observed by the comparative study. Of importance, knockdown of all except PLOD2 reduced the proliferation of HBMECs under normoxic condition. Also, we observed that following 72 h of hypoxia exposure no cells were alive in the control group. In contrast, following Spag4, adrenomedullin, ENO2, and ERO1L knockdown, we observed a significant number of viable cells present following 72 h of hypoxia. However, there was still a considerable amount of cell death in each of the knockout cells, which suggests that multiple gene knockout need to be investigated. The most significant finding of the present study was to observe a 100% cell death following 48 h of hypoxia exposure in EGLN3 knockout cells.

### Sperm associated antigen 4 and chronic hypoxia

Sperm associated antigen 4 (SPAG4) was identified in sperm tail and is known to play a crucial role in sperm motility (Tarnasky et al., 1998; Shao et al., 1999). Notably, the recent studies have also implicated SPAG4 in cell cycle regulation (Shoji et al., 2013). The upregulation of SPAG4 with chronic hypoxia may be crucial in both cell-cycle arrest and cell migration which are critical steps in hypoxia-induced angiogenesis and other acclimatization responses.

### Adrenomedullin and chronic hypoxia

Adrenomedullin is one of the molecules, which was altered with chronic hypoxia in each of the microarray studies analyzed. On conducting a PubMed search with keywords “Adrenomedullin” and “Hypoxia” in the Title/Abstract of the studies indexed, we found 198 studies in which adrenomedullin was upregulated in response to hypoxia. Adrenomedullin was discovered in 1993 from human pheochromocytoma and was regarded as one of the major circulating vasodilator peptides with therapeutic potential. Adrenomedullin is secreted by many cells and has well established smooth muscle relaxing property. It may play an important role in the hypoxia-induced increase in blood flow. Moreover, hypoxia-induced adrenomedullin is implicated in various processes including inhibition of apoptosis-mediated cell death, tumor metastasis, angiogenesis, and inhibition of immune surveillance (Larrayoz et al., 2014). Based on adrenomedullin upregulation in hypoxia, and its effect on angiogenesis as well as prevention of apoptosis, studies have been conducted to evaluate its usefulness in cancer treatment. Several animal studies have demonstrated that inhibition of adrenomedullin leads to a reduction in tumor growth and size. Furthermore, adrenomedullin has been shown to be protective against hypoxia-induced pulmonary vascular remodeling (Matsui et al., 2004). However, there are no studies utilizing adrenomedullin over-expression in stroke or another brain ischemia/hypoxia model. Furthermore, adrenomedullin is predicted to be a HIF1A responsive gene. However, others and we have observed that with chronic hypoxia HIF1A is not upregulated. Thus, it is not clear what maintains the upregulated transcription of adrenomedullin. Other conditions in which adrenomedullin is upregulated include the administration of cytokines such as tissue necrosis factor alpha, interleukin 1, angiotensin II, endothelin, and neurotransmitter such as the atrial natriuretic peptide (Beltowski and Jamroz, 2004). It also is upregulated by hyperglycemia (Beltowski and Jamroz, 2004). Nonetheless, further investigations are needed to identify mechanisms involved in chronic hypoxia-induced adrenomedullin upregulation.

### Chronic hypoxia and SLC2A3

SLC2A3 encodes glucose transporter 3 (GLUT3) involved in facilitated glucose transporter. Homozygous deletion of the gene is embryonic lethal (Schmidt et al., 2008). Under hypoxic stress, cells are more dependent on anaerobic glycolysis and the demand of glucose increases. Thus, upregulation of SLC2A3 is crucial to prevent relative glucose deprivation. Moreover, studies have demonstrated that under hypoxic condition HIF1 enhances the interaction of histone lysine (K)-specific demethylase 3A (KDM3A) to the SLC2A3 promoter and modifies the chromatin conformation to upregulate SLC2A3 expression. This may explain the upregulation of SLC2A3 and other HIF1A regulated genes even when HIF1A levels return to normal. Moreover, KDM3A is a dioxygenase with Jumonji C domain-containing histone demethylation protein whose activation is dependent on cellular oxygen levels. Clearly, the mechanisms are not completely understood.

### Chronic hypoxia and endoplasmic oxidoreductin-1 like protein

Second to mitochondria, the endoplasmic reticulum (ER) is the highest user of molecular oxygen. In the ER, newly translated proteins undergo post-translational modifications, such as folding, disulfide bond formation, glycosylation etc. These modifications are important in proper function and long-term stability of the proteins. ERO1L protein acts as a catalyst for oxidative disulfide bridge formation and plays an important role in oxidative protein folding and ER stress. This protein is known to be upregulated with hypoxia in rats, mice, and humans (Gess et al., 2003). Thus, when the oxygen levels are reduced, ERO1L act as an important mechanism to prevent protein misfolding and ER stress. ERO1L is a known transcriptional target of HIF1A; however, it remains unclear that how it remains elevated despite reduced HIF1A under chronic hypoxic conditions. However, other mechanisms that lead to upregulated ERO1L are adipogenesis and ER stress resulting from factors other than hypoxia. During adipogenesis, peroxisome proliferator-activated receptor gamma (PPARG) has been involved in ERO1L upregulation. Similarly, during ER stress C/EBP homologous protein (CHOP) is known to bind to its promoter. Both PPARG and CHOP may be involved in hypoxia-induced ERO1L upregulation; however, investigations are needed to examine these possibilities.

### Chronic hypoxia and collagen synthesis and crosslinking

Hypoxia is known to induce collagen synthesis and crosslinking (Horino et al., 2002; Tabima et al., 2012; Liu et al., 2013; Makris et al., 2014). The important steps in collagen synthesis include hydroxylation of proline and lysine by prolyl and lysyl hydroxylases (Smith and Rennie, 2007). In the present study (with several other microarray studies analyzed), we observed a significant upregulation of both prolyl hydroxylase (P4HA1) and lysyl hydroxylase (PLOD2). Lysyl hydroxylase has been shown to be upregulated in whole blood in patients with severe sleep apnea (Perry et al., 2013). Similarly, several other studies have demonstrated that hypoxia induces prolyl and lysyl hydroxylase upregulation (Brinckmann et al., 2005; Eisinger-Mathason et al., 2013; Gilkes et al., 2013). Following collagen synthesis, the next major step is the crosslinking of the newly formed collagen strands. In the present study, we identified lysyl oxidase (LOX) to be significantly upregulated with chronic hypoxia. Lysyl oxidase plays an important role in collagen crosslinking (Makris et al., 2014). Importantly, hypoxia-induced LOX upregulation has been shown to be beneficial for cardiovascular tissue engineering (Van Vlimmeren et al., 2010) as well as engineered articular cartilage (Makris et al., 2015). Furthermore, in the microarray studies with more than 48 h of hypoxic exposure, we observed a significant upregulation of collagen, type V, alpha 1 (COL5A1). Moreover, several other studies also demonstrate upregulation of COL5A1 with chronic hypoxia (Evens et al., 2010; Ramirez et al., 2012). Importantly, COL5A1 forms collagen 5, which is a regulatory fibril-forming collagen critical for fibrillogenesis (Sun et al., 2011). Based on these findings it is apparent that chronic hypoxia leads to upregulation of collagen synthesis, which may be critical in hypoxia-induced angiogenesis.

### Chronic hypoxia and DNA methylation

Evidence suggests that HIF1A rise initially with hypoxia, but it degrades with continued hypoxia, and may not be the master regulator of chronic hypoxia (Ginouvous et al., 2008; Watson et al., 2009; Goyal and Longo, 2014). However, HIF1A is critical for cell survival in the initial phase of hypoxia (acute hypoxia; Tanaka et al., 2011). HIF1A may lead to epigenetic changes, which persist even after HIF1A is no longer upregulated with continued hypoxic stress. The present study demonstrates that chronic hypoxia is associated with changes in DNA methylation. However, only hypermethylation changes were correlating with downregulated genes, and there was no correlation between hypomethylation and upregulated genes. Similarly, another genome-wide methylation study using a short-term (24 h) hypoxia (1% O_2_) demonstrated very few genes were correlating with changes in DNA methylation (Hartley et al., 2013). Thus, it appears that DNA methylation may not be the major mechanism regulating chronic hypoxia-mediated changes in the transcriptome.

### EGLN3 is crucial for cell survival under hypoxic conditions

Crispr/Cas9-mediated knockdown of EGLN3 has the most dramatic effect on cell survival under hypoxic conditions. Importantly, there was a significant effect of EGLN3 knockout on cell viability under hypoxic conditions even during first 24 h. EGLN3 is the chief isoforms, which hydroxylate HIF1A and mark it for degradation. Under hypoxic conditions, EGLN3 is unable to hydroxylate HIF1A and leads to its accumulation. We observed a significant upregulation of EGLN3 expression with hypoxia, which might be crucial for balancing HIF1 pathway over activation. Similar findings of EGLN3 upregulation have been demonstrated by several other studies. Moreover, the role of HIF1A is not well established in EGLN3 upregulation under hypoxic condition. Several studies have demonstrated that EGLN3 upregulation is dependent on HIF1A (Del Peso et al., 2003; Marxsen et al., 2004), whereas other studies have demonstrated that EGLN3 upregulation is independent of HIF transcription factors (Tanaka et al., 2014). Moreover, on comparing the three studies which predicted HIFA targeting genes (Benita et al., 2009; Mole et al., 2009; Ortiz-Barahona et al., 2010) EGLN3 was not identified as one of the HIF1A regulated genes. Furthermore, EGLN3 has been shown to be upregulated in a number of cancers, which show significant tolerance to hypoxic insult. Thus, it appears that EGLN3 will be an excellent target for hypoxia-tolerant tumors.

## Conclusion and perspective

In the present study, we have identified a core group of 9 genes, which were upregulated with continued hypoxia in a number of human cell lines. Importantly, EGLN3 is the most crucial gene for cell survival under hypoxic conditions and may prove to be an excellent target to kill hypoxic tumor cells. Additionally, Spag4, adrenomedullin, ENO2, ERO1L and LOX knockdown were able to provide a relative protection against hypoxia and may be useful to protect cell death under hypoxia-ischemia pathologies including heart disease and stroke. Moreover, the exact mechanisms of ERO1L, LOX, and other genes upregulated despite normal levels of HIF raises the possibility of the HIF-independent mechanism involved in cell survival under prolonged hypoxia.

## Author contributions

RG: conception and study design; EM, DG, and RG: acquisition, analysis and interpretation of data, writing of manuscript, final approval, and agreement to be accountable for all aspects of the work.

### Conflict of interest statement

The authors declare that the research was conducted in the absence of any commercial or financial relationships that could be construed as a potential conflict of interest.

## Supplementary material

Supplementary Figure 1 Figure demonstrates the total RNA quality analysis blot.

Supplementary Figure 2 Figure demonstrates the cRNA quality analysis. **(A)** Blot showing adequate fragmentation of cRNA, and **(B)** cRNA electropherograms of the 12 samples.

Supplementary Figure 3 Figure demonstrate the Scatter Plot Analysis of the probes which demonstrated intensity above background in at least 4 replicates following hypoxia treatment (21,221–21,938 probes). Each spot is the mean of 6 replicate samples. Intensity values are normalized to the 75th percentile intensity of each array. Diagonal lines indicate 2-fold differential expression. Red/Orange, High expression; Yellow, Medium expression; Blue, Low expression.

Supplementary Figure 4 Figure demonstrates the Condition and Gene Tree Clustering of the differentially expressed genes (>2-fold, *T*-test *p*-value < 0.05, 6,328 probes) control vs. hypoxia, which were normalized to median expression across 12 samples. Red/Orange, High expression; Yellow, Medium expression; Blue, Low expression.

Supplementary Figure 5 Figure demonstrates the exemplary trace of the High Resolution Melting Curve Analysis of EGLN3 gene knockout.

Supplementary Figure 6 **(A)** Demonstrates the cobble-stone appearance of human brain micro-endothelial cells. **(B)** Demonstrates capillary tube formation on plating the endothelial cells on Matrigel. **(C)** Demonstrates the green staining of HBMEC to FITC conjugated Ulex Lectin antigen, and **(D)** shows red staining to CD31 antigen.

Supplementary Table 1 Table lists the crispr gRNA used in the present study.

Supplementary Table 2 Table lists the primers used in the present study.

Supplementary Table 3 Table lists the genes upregulated by hypoxia exposure.

Supplementary Table 4 Table lists the genes downregulated by hypoxia exposure.

Supplementary Table 5 Genes common to three studies analyzed to identify HIF-responsive genes.

Supplementary Table 6 Genes hypermethylated with chronic hypoxia exposure.

Supplementary Table 7 Genes hypomethylated with chronic hypoxia exposure.

## Abbreviations

ADM: Adrenomedullin
ASMC: Aortic smooth muscle cells
CAS9: CRISPR associated endonuclease
CHOP: C/EBP homologous protein
CM: Cardiomyocytes
COL5A1: Collagen, type V, alpha 1
CpG: Clustered regularly interspaced short palindromic repeats
CRISPR: Cytosine guanine phosphodiesters nucleotides
Crna: Complimentary RNA
EGLN3: Egg laying defective-9 family hypoxia-inducible factor 3
ENO2: Enolase 2
ER: Endoplasmic reticulum
ERO1L: ERO1-like (*S. cerevisiae*)
GLUT3: Glucose transporter 3
Grna: Guide RNA
HBMEC: Human brain microendothelial cells
HC: Primary hepatocytes
HIF1A: Hypoxia inducible factor 1 alpha
HUVEC: Human umbilical vein endothelial cells
IHC: Immunohistochemistry
KDM3A: Histone lysine (K)-specific demethylase 3A
LEC: Lymphatic endothelial cells
LOX: Lysyl oxidase
MCF4: Breast carcinoma cell line
P4HA1: Prolyl 4-hydroxylase, alpha polypeptide I
PBL: Peripheral blood lymphocytes
PHD: Prolyl hydroxylase deoxygenase
PLOD2: Procollagen-lysine, 2-oxoglutarate 5-dioxygenase 2
PPARG: Peroxisome proliferator-activated receptor gamma
PVMEC: Pulmonary vein microendothelial cells
REC: Renal epithelial cells
SLC2A3: Solute carrier family 2 (facilitated glucose transporter), member 3
SPAG4: Sperm associated antigen 4
TR: Transcriptional Regulator.
