# Supplementary figures and images for: Comparative and Experimental Studies on the Genes Altered by Chronic Hypoxia in Human Brain Microendothelial Cells
Source: Front Physiol. 2017 May 31;8:365. doi: 10.3389/fphys.2017.00365 (PMC5450043; doi:10.3389/fphys.2017.00365)

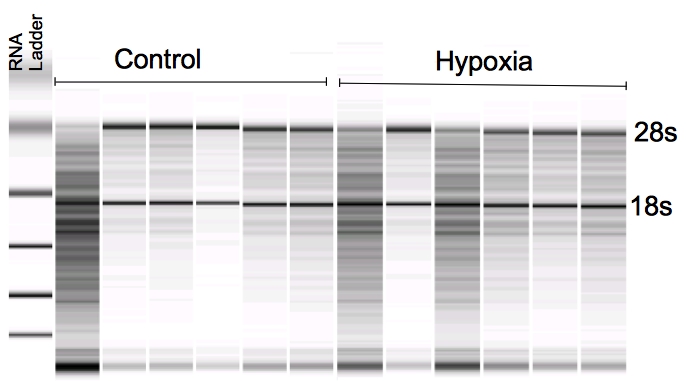

Supplement: Supplementary Figure 1 — Figure demonstrates the total RNA quality analysis blot. [file Image1.JPEG]

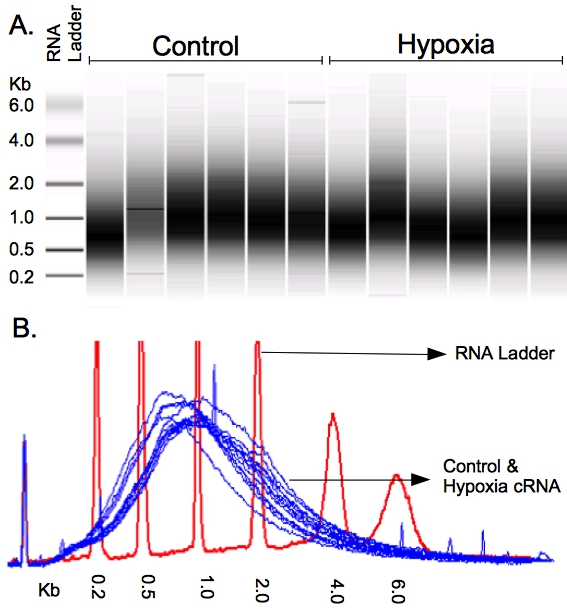

Supplement: Supplementary Figure 2 — Figure demonstrates the cRNA quality analysis. (A) Blot showing adequate fragmentation of cRNA, and (B) cRNA electropherograms of the 12 samples. [file Image2.JPEG]

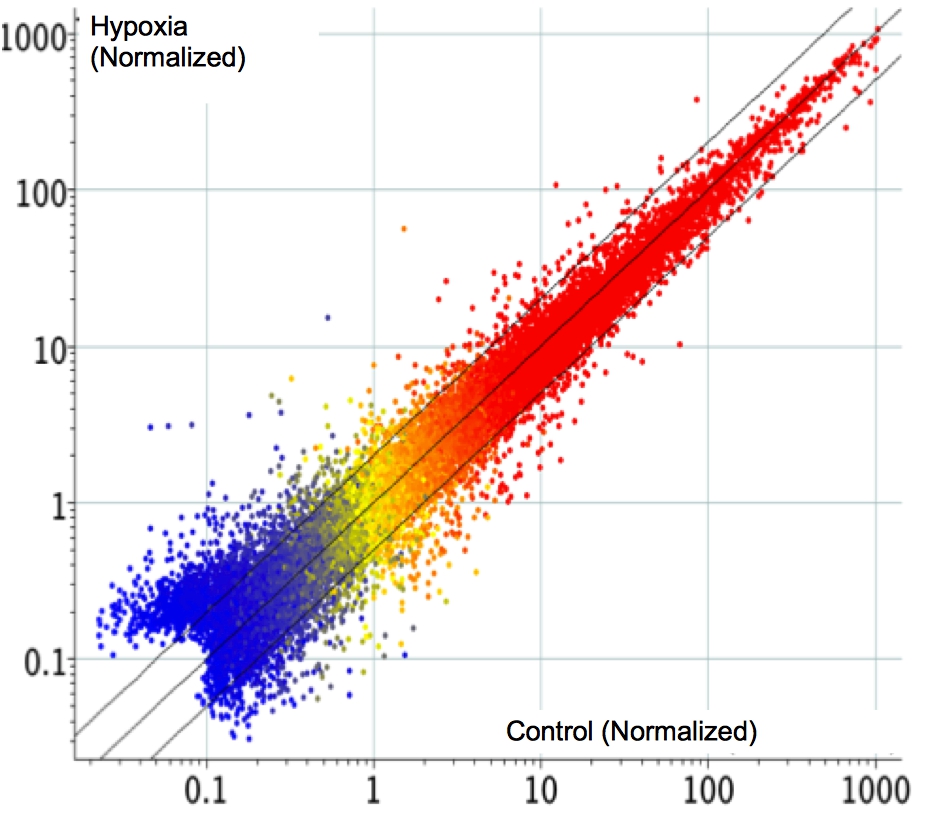

Supplement: Supplementary Figure 3 — Figure demonstrate the Scatter Plot Analysis of the probes which demonstrated intensity above background in at least 4 replicates following hypoxia treatment (21,221–21,938 probes). Each spot is the mean of 6 replicate samples. Intensity values are normalized to the 75th percentile intensity of each array. Diagonal lines indicate 2-fold differential expression. Red/Orange, High expression; Yellow, Medium expression; Blue, Low expression. [file Image3.JPEG]

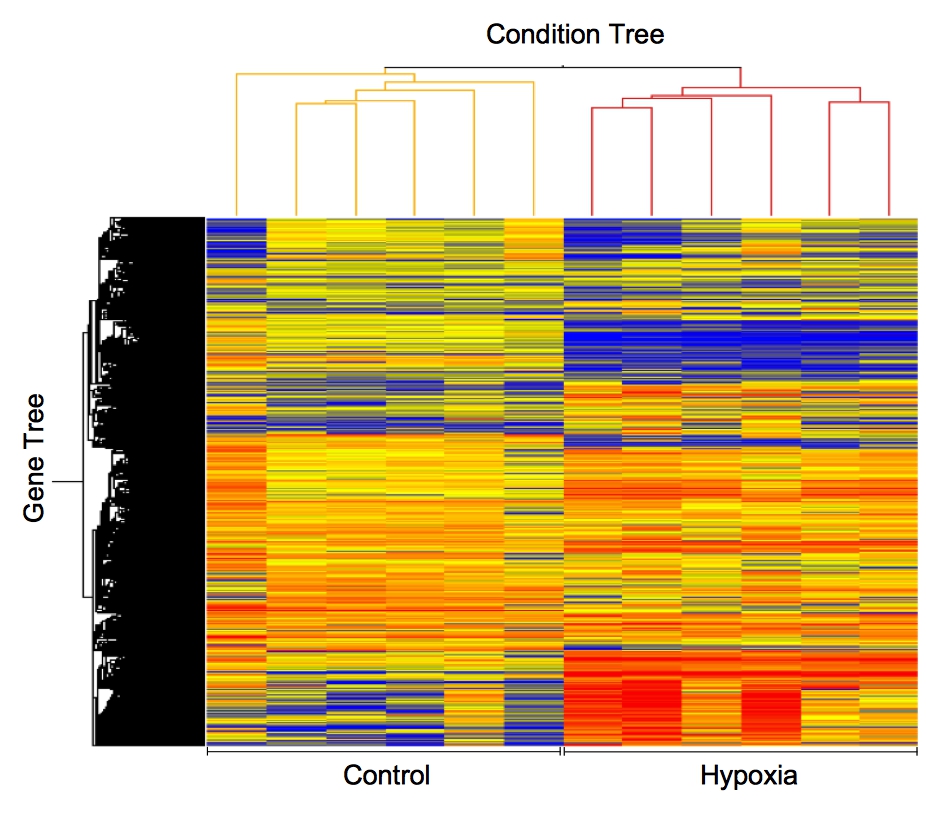

Supplement: Supplementary Figure 4 — Figure demonstrates the Condition and Gene Tree Clustering of the differentially expressed genes (>2-fold, T-test p-value < 0.05, 6,328 probes) control vs. hypoxia, which were normalized to median expression across 12 samples. Red/Orange, High expression; Yellow, Medium expression; Blue, Low expression. [file Image4.JPEG]

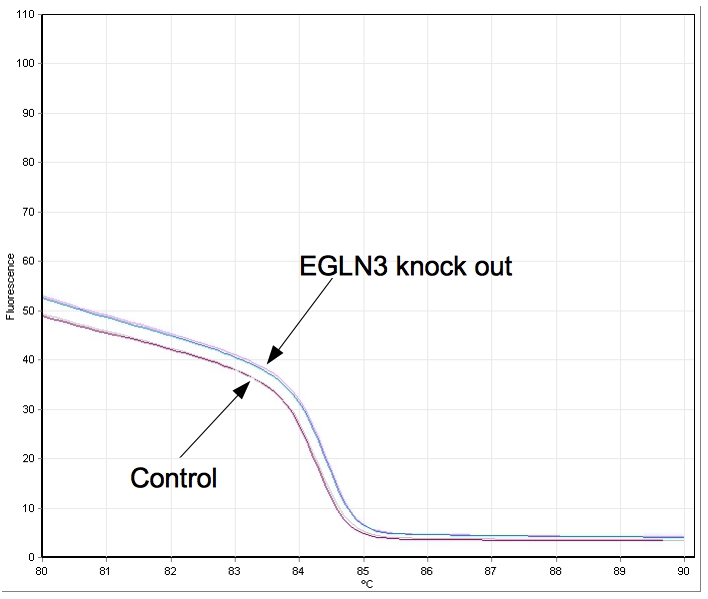

Supplement: Supplementary Figure 5 — Figure demonstrates the exemplary trace of the High Resolution Melting Curve Analysis of EGLN3 gene knockout. [file Image5.JPEG]

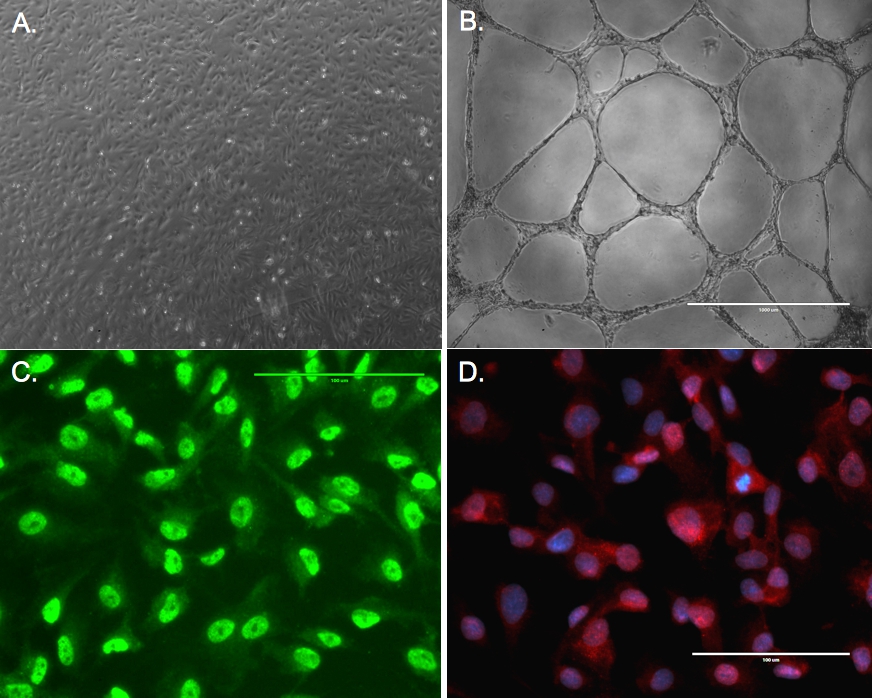

Supplement: Supplementary Figure 6 — (A) Demonstrates the cobble-stone appearance of human brain micro-endothelial cells. (B) Demonstrates capillary tube formation on plating the endothelial cells on Matrigel. (C) Demonstrates the green staining of HBMEC to FITC conjugated Ulex Lectin antigen, and (D) shows red staining to CD31 antigen. [file Image6.JPEG]
